# Neonatal Hearing Screening Using Wideband Absorbance and Otoacoustic Emissions Measured Under Ambient and Pressurized Conditions

**DOI:** 10.3390/children11111290

**Published:** 2024-10-25

**Authors:** Carolina Verônica Lino Novelli, Milaine Dominici Sanfins, Piotr Henryk Skarżyński, Magdalena Beata Skarżyńska, Thaís Antonelli Diniz-Hein, Maria Francisca Colella-Santos

**Affiliations:** 1 Faculty of Medical Sciences, State University of Campinas, Campinas, SP 13083-887, Brazil; carolvlino@gmail.com (C.V.L.N.); thais.antonelli@puc-campinas.edu.br (T.A.D.-H.); mfcolell@unicamp.br (M.F.C.-S.); 2 Department of Speech-Hearing-Language, Universidade Federal de São Paulo, São Paulo, SP 04044-020, Brazil; 3 Post-Graduate Program in Clinical Audiology, Instituto de Ensino e Pesquisa Albert Einstein, São Paulo, SP 05652-000, Brazil; 4 Department of Teleaudiology and Screening, World Hearing Center, Institute of Physiology and Pathology of Hearing, 05-830 Kajetany, Poland; p.skarzynski@csim.pl; 5 ENT Department, Maria Curie-Skłodowska University, 20-031 Lublin, Poland; 6 MEDINCUS International Hearing and Speech Center, 05-830 Kajetany, Poland; 7 Department of Otolaryngology, Institute of Sensory Organs, 05-830 Warsaw, Poland; 8 Heart Failure and Cardiac Rehabilitation Department, Medical University of Warsaw, 02-091 Warsaw, Poland; 9 World Hearing Center, 05-830 Kajetany, Poland; 10 Department of Pharmacotherapy and Pharmaceutical Care, Medical University of Warsaw, 02-091 Warsaw, Poland; m.skarzynska@csim.pl; 11 Institute of Sensory Organs, 05-830 Kajetany, Poland; 12 Center of Hearing and Speech, 05-830 Nadarzyn, Poland

**Keywords:** neonates, otoacoustic emissions, hearing, absorbance

## Abstract

Background: The objective was to analyze wideband acoustic absorbance and transiently evoked otoacoustic emissions (TEOAEs) from newborns without risk indicators of hearing loss and test the effectiveness of measuring TEOAEs under pressurized ear canal conditions. Methods: Evaluation of 102 newborns from a maternity hospital, who stayed in the well-baby nursery and did not have risk indicators for hearing loss. The procedures involved wideband tympanometry and TEOAEs performed at ambient pressure (AP) and at a pressure corresponding to maximum compliance (PP). Newborns were then divided into three groups according to their AP and PP results: G1 (PASS/PASS), G2 (FAIL/PASS), and G3 (FAIL/FAIL). Results: Comparing the three groups, pressurization improved the pass rate for G2 only. For wideband absorbance, differences were statistically significant for frequencies of 2, 3, and 6 kHz, with lower values under the AP condition. For TEOAEs, the differences were statistically significant in all bands, with lower values under the AP condition. Conclusions: Pressurization was effective in detecting more TEOAEs in G2, thus reducing the number of failures in neonatal hearing screening and reducing the need to return for retesting.

## 1. Introduction

Newborns are born able to speak, but oral language requires auditory experience to emerge. The lack of auditory stimulation, resulting from hearing loss, leads to a weakening of the synapses that should occur for the proper development of speech. Thus, the normal development of oral language requires, among other factors, an intact auditory system. Congenital hearing loss affects 2–4 neonates among 1000 live births in the rooming-in ward [1]. Early detection and intervention programs for hearing loss have a positive impact on the auditory development of infants with congenital hearing loss, and neonatal hearing screening (NHS) is the first step in identifying hearing loss as soon as possible [2]. The capture of otoacoustic emissions (OAEs) has been used in NHS programs mainly because it is a fast, objective, and effective method of differentiating children with hearing thresholds within normal limits from those with a degree of hearing loss [3].

It is recommended that NHS of newborns who do not present risk indicators for hearing loss be performed using OAEs before hospital discharge; in cases of failure (that is, in the absence of otoacoustic emissions), it is necessary to perform a retest of the newborn within 15 days. If there is again a failure to capture emissions, the newborn is referred for audiological diagnosis using Brainstem Auditory Evoked Potentials (BAEPs) and other procedures.

Transient evoked otoacoustic emissions (TEOAEs) are sounds of specific frequencies generated by the cochlea in response to a repeated train of clicks; the responses are manifested as a complex sound wave. The presence of strong OAEs generally indicates healthy outer hair cells (OHC), although their absence does not necessarily mean defects in the inner ear since OHCs also depend on the integrity of the outer and middle ear. There are reports of limitations to current TEOAE procedures and there is still room for optimization [4].

The pressure in the middle ear (ME) of the newborn may be different from the pressure in the outer ear due, for example, to the immaturity of the Eustachian tube or the presence of embryonic connective tissue, mesenchyme, or other materials [5]. Since the middle ear is important for transmitting the stimulus to the cochlea, as well as for capturing the OAEs returning through the middle ear to the external acoustic meatus (EAM), a dysfunction in this pathway, including tension of the tympanic membrane caused by pressure, can attenuate the signal and prevent TEOAEs from being captured. Thus, the proper functioning of all the middle ear structures is essential for correct stimulation and measurement of OAEs [5].

Using wideband tympanometry (WBT), it is possible to make several measurements of the auditory system, including its wideband absorbance (WBA). Studies have shown that, compared to 1 kHz tympanometry, WBT is more accurate in detecting newborns with anomalies in sound conduction [6,7]. Keefe et al. [8] have shown that WBT responses allow for improved analysis of OAEs when middle ear dysfunction is present, and its use in NHS can improve subsequent diagnosis of hearing loss.

It is now possible to perform OAE and WBT measurements using the same equipment and, at the same time, equalize the pressure between the EAM and the middle ear, allowing OAEs to be captured at the maximum middle ear compliance. Given the documented negative impact of middle ear alterations on the detection of OAEs and the high prevalence of altered middle ear pressure in newborns, it appears to be important to consider compensating for middle ear pressure when measuring OAEs. Pressure compensation can result in increased detection of TEOAEs, which makes it possible to confirm the normal functioning of outer hair cells in a greater number of cases than when there is deviant middle ear pressure. This is important for NHS where the absence of TEOAEs can mean a delay in providing information about the child’s hearing to the family; in addition, a greater number of retests in hearing screening leads to a delay in detecting real cases of hearing loss.

This study investigates the use of pressurized OAEs in NHS. The aim is to test the effectiveness of pressurizing otoacoustic emissions in improving the results of screening, thereby reducing the number of return visits needed.

## 2. Materials and Methods

### 2.1. Ethics

This is a descriptive, cross-sectional, prospective observational study approved by the Research Ethics Committee of Faculty of Medical Sciences, Unicamp, under opinion no. 932.602. Since the work was conducted in a public maternity hospital in the state of São Paulo, Brasil, and located in a teaching hospital, the mother gave free and informed consent and signed the form requesting participation in research at the time of admission.

### 2.2. Participants

The study participants were 102 newborns, 44 females and 58 males, admitted to the maternity rooming-in ward between October 2017 and November 2021, where they participated in NHS before discharge. The age of the newborns at the time of the evaluation was 1 to 4 days of life, with an average of 2.19 days of life. The mothers and babies were volunteers and did not receive any compensation for their participation.

### 2.3. Inclusion Criteria

Individuals qualified to participate in the research were newborns born between October 2017 and November 2021. They remained in joint accommodation with good health conditions at birth and without risk indicators for hearing loss as indicated by the Joint Committee on Infant Hearing [2].

### 2.4. Exclusion Criteria

The exclusion criteria for the present study were the following: structural alterations of the external ear in at least one ear or genetic syndromes associated with cranial malformation.

### 2.5. Procedures

The selected newborns, who were in natural sleep, were taken to the hospital’s acoustically treated room for the assessments. The assessment procedures were performed using the Titan/Interacoustics (Middelfart, Dinamarca) equipment, connected to a laptop, and interfaced with the Titan Suite software, version 3.4.0. The Titan probe contained two receiver ports and a microphone port; the acoustic responses were recorded by the probe microphone, amplified, and digitized. An air pressure pump was coupled to the tympanometer and allowed for computer control of the probe pressure sweep; a pressure sensor, accurate to 1 daPa, was located between the pressure pump and the ear probe. When recording acoustic responses, air pressures were read by the sensor every 25 ms and delivered to the computer, which determined the required pressure sweep speed and sent commands to the digital signal processor via the serial port for adjustment of the pressure [9].

#### 2.5.1. Wideband Tympanometry (WBT)

The probe was inserted into the newborn’s EAM using an appropriate olive, and a click stimulus was presented at 96 dB sound pressure level (SPL) while the air pressure was swept from +200 to −300 daPa. A microphone inside the probe captured the stimulus returning to the EAM, and based on the relationship with the presented stimulus, the internal software calculated the WBA. This way, a three-dimensional graph was generated with the following axes: frequency from 226 to 8000 Hz, pressure, and WBA.

Two WBA settings were selected for analysis: first, the WBA at ambient pressure (AP), and second, the WBA at a pressure corresponding to peak compliance (PP). The peak pressure value was calculated by the equipment’s software, which considered the mean of the tympanograms obtained between 800 and 2000 Hz, the frequency range used for newborns.

#### 2.5.2. Transient Otoacoustic Emissions (TEOAEs)

After the WBT measurement was performed and without moving the probe in the EAM, the TEOAE was captured with the EAM at ambient pressure (AP). The collection was accomplished with 300 sweeps, in one ear at a time, with a click-type stimulus, at 83 dB SPL peak equivalent.

For data analysis, the signal-to-noise (S/N) ratios of the TEOAEs were considered in the range 0.5 to 5.5 kHz, with center frequencies of 0.87, 1.94, 2.96, 3.97, and 4.97 kHz. The S/N ratio compares the level of a desired signal to the background noise level; it is defined as the relationship between the signal power and the noise power expressed in decibels and expresses the difference between the measured OAE and the background noise level.

The WBA analysis was performed in the frequency bands of 1, 2, 3, 4, and 6 kHz; the selection of the analyzed frequencies, within the absorbance frequency range provided by the equipment, was made so that the WBA data could be compared with TEOAEs in the same frequency range.

The criteria for the presence of a PASS level of TEOAE were as follows: overall reproducibility ≥50%, probe stability ≥70%, and S/N ratio per frequency band ≥6 dB, with mandatory presence in at least three frequency bands, as used by several studies [10,11,12]. In the absence of these responses, the TEOAE was deemed to be absent (FAIL).

After capturing the TEOAE at ambient pressure, the measurement was performed under pressurized conditions (PP)—at the pressure where the compliance was at its maximum and obtained at the same time as the WBT.

In some cases, it was not possible to measure OAEs in both ears due to factors such as movement or crying of the newborn, and these ears were excluded from analysis. The mean time to complete a full evaluation in the same newborn—with WBT and TEOAE in both AP and PP stages—was around 10 min.

Based on the capture of TEOAE at ambient pressure and peak pressure, it was possible to divide the newborns into three groups:

G1: newborns (ears) with a PASS result under the ambient pressure condition and a PASS result under the peak pressure condition;

G2: newborns (ears) with a FAIL result under the ambient pressure condition and a PASS result under the peak pressure condition;

G3: newborns (ears) with a FAIL result under the ambient pressure condition and a FAIL result under the peak pressure condition.

This division allowed us to analyze the results of absorbance and amplitude of TEOAE in homogeneous groups and compare the difference in results for each test condition and between groups. In addition, at the conclusion of the study, we were able to refer newborns after screening based on the results obtained.

### 2.6. Statistical Analysis

Statistical analysis was performed using a specially prepared data entry program and sent for statistical analysis by a professional in the area who used the statistical analysis system for Windows, version 9.2 (SAS Institute, Cary, NC, USA).

To compare categorical variables between groups, the chi-square or Fisher’s exact tests (for expected values less than 5) were used. To compare numerical variables, the Mann–Whitney test (2 categories) and the Kruskal–Wallis test (3 categories) were used due to the absence of a normal distribution of the variables. The significance level adopted for the statistical tests was 5% (i.e., *p* < 0.05), and values are highlighted here in bold.

## 3. Results

### 3.1. Descriptive Results of the Sample, Considering the Right Ear (RE) and Left Ear (LE)

A total of 102 newborns were evaluated, 44 females and 58 males, from October 2017 to November 2021. Of the 102 newborns evaluated, 83 had both ears evaluated, 9 had only the right ear evaluated, and 10 had only the left ear evaluated, totaling 185 ears. Table 1 shows the distribution of the sample, considering the ear evaluated and gender.

### 3.2. TEOAE Results Obtained at Ambient Pressure

Table 2 shows the newborns (ears) distributed according to the PASS or FAIL result, obtained for the TEOAEs captured under the AP condition.

Table 3 presents the means and standard deviations of S/N of TEOAE in each group, in the AP condition.

A statistically significant difference (*p* < 0.05) was observed between the groups for all frequency bands tested, with lower values for the S/N ratio obtained in the group of newborns with a FAIL result.

After performing the TEOAE at ambient pressure, the TEOAE test was performed under the PP condition. Based on the comparison of the PASS/FAIL results obtained under both conditions (AP/PP), we grouped the newborns (ears) into three groups:

G1 (*n* = 127 ears—68.6%): PASS under the AP condition/PASS under the PP condition;

G2 (*n* = 22 ears—11.9%): FAIL under the AP condition/PASS under the PP condition;

G3 (*n* = 36 ears—19.5%): FAIL under the AP condition/FAIL under the PP condition.

### 3.3. Results of the TEOAE and WBA Variables, Considering Groups G1, G2, and G3

When analyzing the ear variable in G1, it was found that there was no ear effect between the RE (right ear) and LE (left ear) in the TEOAE results. For this reason, the data from both ears were combined. The results of the analyses performed considering the division into groups under the AP and PP conditions are shown below.

#### 3.3.1. G1—PASS/PASS

**Figure 1:**
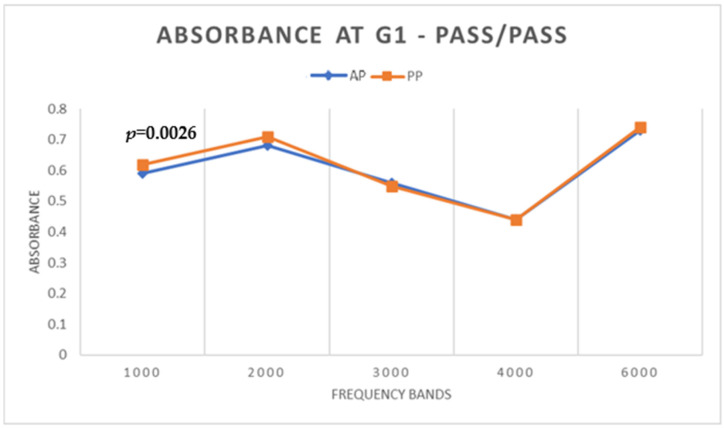
Mean WBA values under AP and PP conditions in G1.

In G1, with regard to WBA, there was a statistically significant difference (*p* < 0.05) only at the frequency of 1000 Hz (Figure 1), with a lower absorbance value under the AP condition.

**Figure 2:**
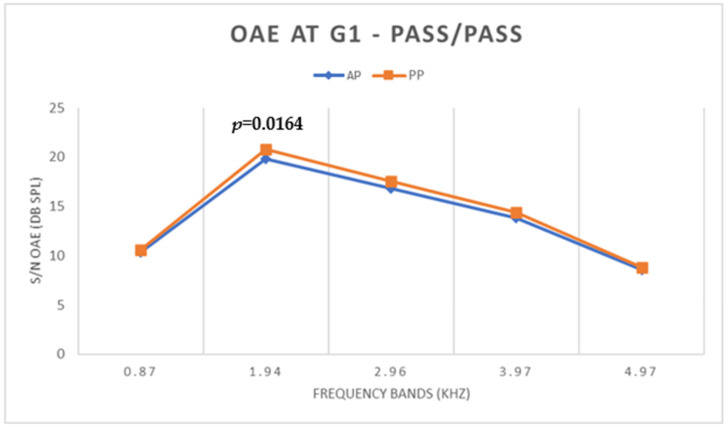
Mean S/N values of TEOAE under AP and PP conditions in G1.

Regarding TEOAE, there was a statistically significant difference (*p* < 0.05) only in the 1.94 kHz frequency band (Figure 2), with a lower TEOAE S/N value under the AP condition.

#### 3.3.2. G2—FAIL/PASS

**Figure 3:**
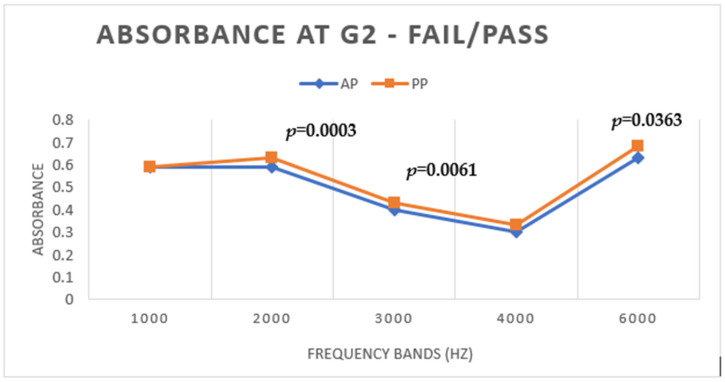
Mean WBA values under AP and PP conditions in G2.

In G2, in relation to WBA, there was a statistically significant difference (*p* < 0.05) for frequencies of 2, 3, and 6 kHz, with the lowest values under the AP condition (Figure 3).

**Figure 4:**
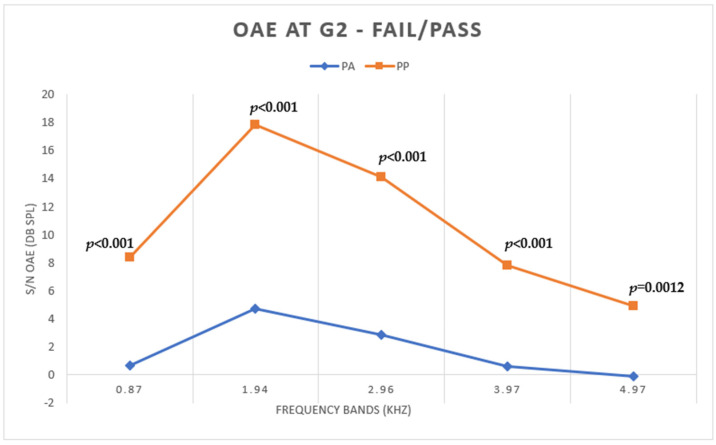
Mean S/N values of TEOAE under AP and PP conditions in G2.

Regarding TEOAE, there was a statistically significant difference in the S/N ratio between all frequencies analyzed, with the lowest values under the AP condition (Figure 4).

#### 3.3.3. G3 FAIL/FAIL

**Figure 5:**
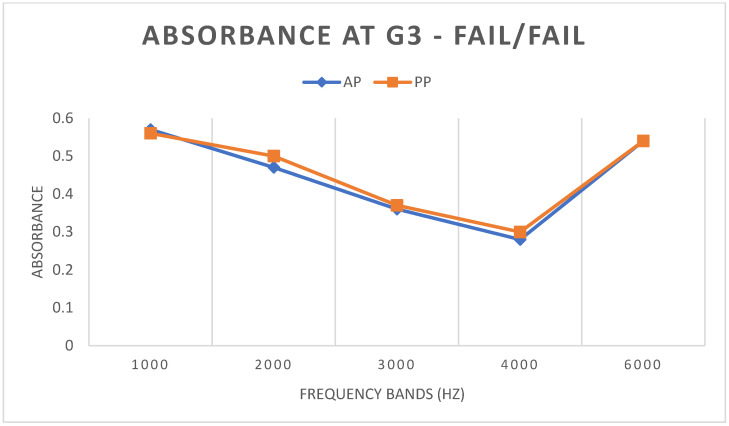
Mean WBA values in G3 under AP and PP conditions.

**Figure 6:**
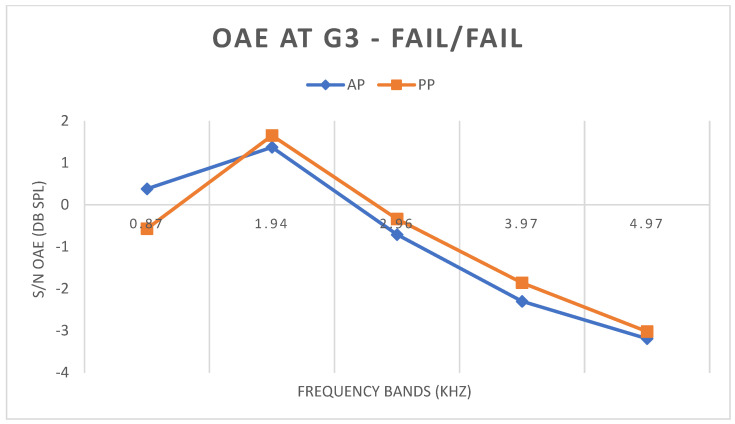
Mean S/N values of TEOAE in G3 under AP and PP conditions.

In G3 FAIL/FAIL, both in terms of WBA and TEOAE, there was no statistically significant difference in any band between the AP and PP conditions (Figure 5 and Figure 6).

### 3.4. Analysis of the Comparison of the Results Between Groups

**Figure 7:**
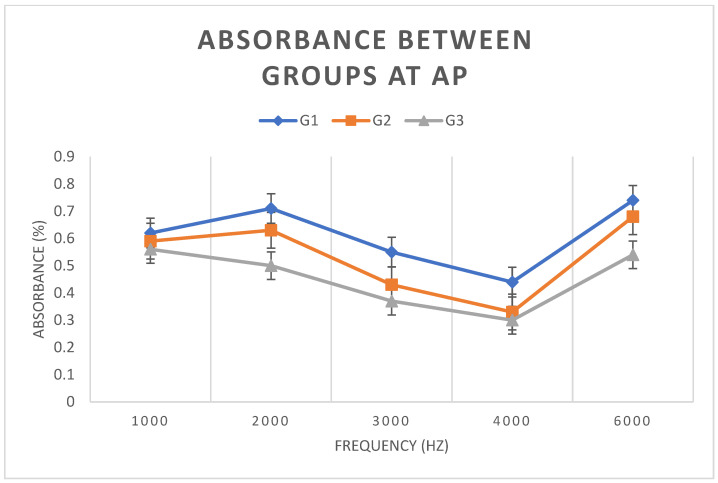
Means and standard deviations of WBA for groups G1, G2, and G3, under the AP condition.

Means and standard deviations of absorbance in each group under the AP condition were analyzed. A statistically significant difference (*p* < 0.05) was observed between groups G1 and G2 at frequencies of 2, 3, and 4 kHz (*p* < 0.0035). Similarly, there was a statistically significant difference between groups G1 and G3 at frequencies of 2, 3, 4, and 6 kHz (*p* < 0.001). However, there was no statistically significant difference between groups G2 and G3 (*p* > 0.25). Figure 7 shows the mean and standard deviation values of WBA in each group at AP, showing that G2 presented intermediate values between G1 and G3 at all frequencies.

**Figure 8:**
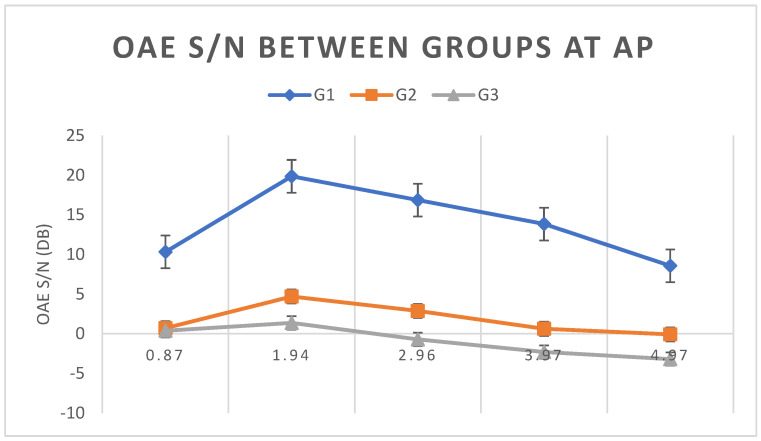
Means and standard deviations of TEOAE S/N values in groups G1, G2, and G3 under the AP condition.

The means and standard deviations of S/N values in each group under the AP condition were also calculated. A statistically significant difference (*p* < 0.0001) was observed between groups G1 and G2 and between groups G1 and G3 in all frequency bands tested. However, between groups G2 and G3, the difference was only significant for the bands of 3.97 and 4.97 kHz (*p* < 0.01). Figure 8 shows the means and standard deviations of S/N of TEOAE in each group under the AP condition. It is evident that the S/N ratio for G1 is much greater than for G2 and G3.

**Figure 9:**
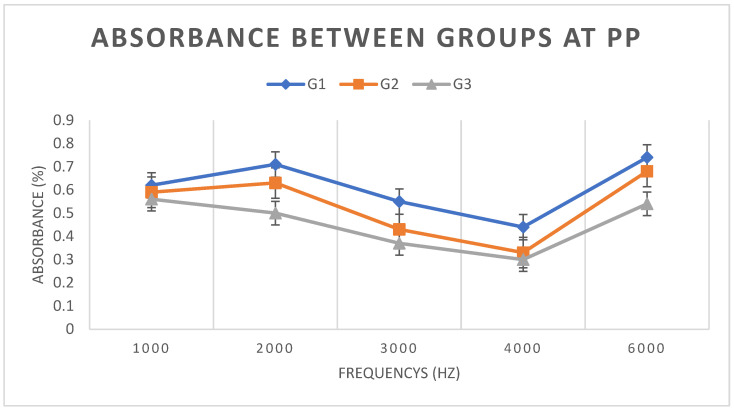
Means and standard deviations of WBA for groups G1, G2, and G3 under the PP condition.

Turning to absorbance values, the means and standard deviations of WBA in each group under the PP condition were calculated. There was a statistically significant difference between groups G1 and G2 at frequencies of 2, 3, and 4 kHz (*p* < 0.05) and between groups G1 and G3 at frequencies of 2, 3, 4, and 6 kHz (*p* < 0.005), and between groups G2 and G3 only at 2 kHz (*p* < 0.01). Figure 9 shows the means and standard deviations of WBA in each group under the PP condition.

**Figure 10:**
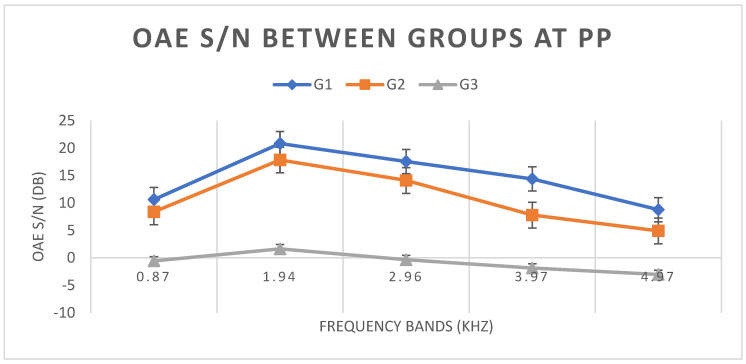
Means and standard deviations of S/N of TEOAE in groups G1, G2, and G3 under the PP condition.

Means and standard deviations of TEOAE S/N values in each group under the PP condition were calculated. A statistically significant difference was observed between groups G1 and G2 for frequencies of 2, 3, 4, and 6 kHz (*p* < 0.005). Similarly, there was a significant difference between groups G1 and G3 and between G2 and G3 for all frequency bands tested (*p* < 0.0001). Figure 10 shows the means and standard deviations of TEOAE S/N values in each group under the PP condition.

## 4. Discussion

Comparing WBA under the AP and PP conditions, it was found that, in G1 (the group of newborns who passed both tests), there was a statistically significant difference at the lowest frequency of 1000 Hz. At this frequency, the lowest WBA values were obtained under the AP condition. Hunter et al. [9] stated that immature absorbance patterns were more apparent at low frequencies from birth to one month of age—as with the newborns evaluated in this study—and changed substantially to a more mature pattern at 6 months of age.

**Figure 11:**
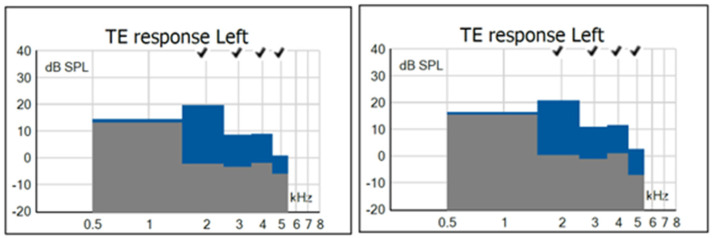
Examples of TEOAE findings in one subject from G1 who passed under AP conditions (**left**) and under PP conditions (**right**). Legend: columns in grey: absence of TEOAE; columns in blue: presence of TEOAE; √: PASS result.

Regarding TEOAEs, in G1, there was a statistically significant difference only in the 1.94 kHz band, with a lower TEOAE S/N value under the AP condition. Figure 11 shows an example in which one can observe that, under both test conditions of ambient pressure and peak pressure, the subject presented a PASS result.

For low frequencies, the difference in WBA between AP and PP did not influence the TEOAE result since these were mostly captured in higher frequency bands (1.94, 2.96, 3.97, and 4.97 kHz) where there were no significant differences in WBA. For TEOAE S/N values, the absence of statistical differences between the AP and PP conditions for most frequencies can be explained by the fact that this is a homogeneous group with PASS results under both conditions. Although pressurization had a positive influence on TEOAE amplitudes at 1.94 kHz, the difference had no effect on the result, indicating that for this group of newborns, pressurization was not required. Thus, newborns who pass screening without the need for pressurization can, in the absence of risk indicators, be discharged since the standard WBT and OAE procedures are adequate to detect a normal middle and inner ear function.

However, for G2, the group of newborns who failed the AP stage and passed the PP stage, the difference in WBA was statistically significant for 2, 3, and 6 kHz, with the lowest WBA values found under the AP condition. Hunter et al. [13] also found that newborns who do not pass OAE screening at birth also have lower absorbance for 1 to 3 kHz, suggesting that such screening failures are frequently associated with middle ear problems at birth.

**Figure 12:**
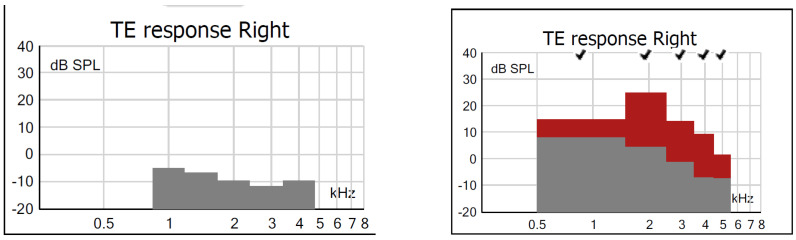
Examples of TEOAE findings in one subject from G2 who failed an AP test (**left**) but passed a PP test (**right**). Legend: columns in grey: absence of TEOAE; columns in red: presence of TEOAE; √: PASS result.

For TEOAEs measured in G2, the differences were statistically significant in all bands, with lower values under the AP condition and higher under PP, showing that lower WBA values improve the capture of TEOAEs. Figure 12 shows that in a newborn from G2, there is a difference between the amplitudes of TEOAEs at AP and PP, and this is representative of newborns who passed under the AP condition and failed under the PP condition.

In studies by Marshall et al. [14], pressurization favored the lower frequency bands (<2 kHz), and the study by Zimatore et al. [15] found differences at all frequencies, with a maximum difference at 4.97 kHz. In our study, however, the highest values were found in the bands beyond 2 kHz (1.94 and 2.96 kHz); however, it should not be forgotten that there are many differences in the application of methods between the studies cited and the current study, which may explain the discrepancy in the results, such as the sample size and age of assessment of newborns.

Changes in energy reflectance and, consequently, in absorbance in normal ears of newborns up to 1 month of age appear approximately in the range of 2 to 5 kHz but are not present at other frequencies [16]. Likewise, Hunter et al. [9] found that newborns in healthy nurseries who failed NHS had significantly lower energy absorbance in the region of 1 to 4 kHz compared with newborns who passed neonatal screening.

The findings of our study indicate that pressurization of the EAM is able to increase the S/N ratios of TEOAEs in all frequency bands tested. This means that many of these subjects were in the FAIL group under the AP condition but, after testing under the PP condition, were part of the PASS group. Such findings demonstrate the effectiveness of pressurization in a number of newborns who initially failed screening. Pressurization adjusts the EAM pressure so as to cancel the pressure difference between the EAM and the middle ear, reducing the rigidity of the system—tympanic membrane and ossicles—and making it possible to capture OAEs.

A major factor contributing to the cost of NHS programs is the high rate of false positives, that is, newborns screened with a failed result that was not confirmed in a retest. Studies have shown that this high rate is due to the inability of current screening methods to distinguish between minor conductive disorders and inner ear disorders, such as sensorineural hearing loss [6,17].

In newborns, the incidence of conductive disorders is approximately 30 times higher than that of inner ear disease [18]. This is because, at birth, the external ear cavity may be obstructed with vernix and the middle ear may be filled with materials such as amniotic fluid, meconium, and epithelial cells. In addition, aeration of the middle ear usually occurs only 48 h after birth [12], meaning that NHS, which is normally performed before the newborn is 2 days old, can be affected by middle ear changes.

According to the NHS flowchart from the JCIH [2], newborns in the NICU might need an automated brainstem auditory evoked response (BAER) test if they fail the OAE test. BAER is a hearing screening exam that aims to evaluate the auditory system as a whole and can show the existence of auditory neuropathy. For newborns in rooming-in where the incidence of auditory neuropathy is very low, it is recommended that NHS be performed by capturing OAE and that, in cases of failure, a reassessment be performed using BAER. This is because BAER is much less affected by middle ear changes than OAEs [19].

However, performing BAER requires greater technical knowledge and skill from the speech-language pathologist when dealing with newborns since it is a test that is much more sensitive to external factors than OAEs. It also requires more time to prepare the newborn and more expense to acquire the necessary material, which, in a public service facility, can be a drawback. For these reasons, in many hospitals, the use of BAER is not feasible, which means that newborns who fail OAE screening are discharged without a second evaluation being performed and have to return for a retest. A false-positive result requiring a retest can generate concern and anxiety in parents and caregivers, in addition to costs resulting from missing a day of work and travel, which can often result in some newborns not having a follow-up if they do not return for reassessment.

Furthermore, it is known that the hearing threshold detected by the BAER technique is slightly higher (40 to 45 dB HL) compared with the TEOAE technique (30 or 35 dB HL) [2]. Thus, in cases of OAE failure, the use of BAER for NHS might mean that hearing loss of around 30 to 45 dB can sometimes be missed.

In our study, the improvement in TEOAE S/N values with the use of pressurization in G2 meant that around 38% of newborns (ears) that had previously failed TEOAE under AP conditions presented a PASS result in PP without the need to perform a new procedure, such as ABR, or return for retesting. We, therefore, suggest replacing ABR with OAE pressurization as the preferred method to reassess newborns who fail the initial screening. This helps avoid failing to detect newborns with hearing thresholds between 30 and 45 dB and reduces the rate of returns for retesting. The reduction in the number of retests also speeds up hearing diagnosis generally since the professional can now focus their efforts on newborns who actually failed, which also helps to reduce the overall cost of NHS programs.

**Figure 13:**
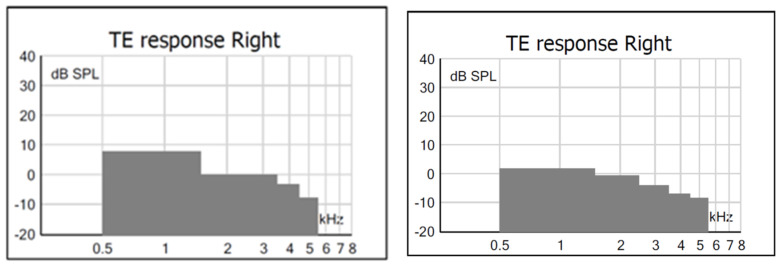
Examples of TEOAE findings in G3 for AP (**left**) and PP (**right**).

Finally, in G3—the group of newborns who failed both stages, both in WBA and TEOAE—there was no statistically significant difference for any frequency tested. The absence of a statistical difference confirms that such newborns will remain in the FAIL group even after PP testing. In Figure 13, for example, it is evident that a subject in G3 presented a FAIL result for TEOAE under both conditions.

Investigations have found that a pressure difference between the middle ear cavity and the EAM attenuates sound transmission through the middle ear and that, for monitoring TEOAE, the pressure in the middle ear should be close to ambient pressure or should be compensated by an equivalent pressure in the EAM [13,18].

Zebian et al. [5] investigated OAEs in cases of alterations in the middle ear and concluded that, with an intact tympanic membrane, it is possible to apply counterpressure to the EAM and improve the admittance of the system.

Hof et al. [18] evaluated 59 children using two OAE measurements, the first at ambient pressure and the second with compensated ME pressure, and reported that the TEOAE response increased by approximately 2 dB under the latter condition. They concluded that equalizing the pressure in the ME by changing the pressure in the EAM can increase the amplitude of TEOAEs, which is especially beneficial for children with negative middle ear pressure.

Finally, Beck et al. [20] concluded that the ability to detect the presence of OAEs in a nonideal ME environment provides a significant advance in diagnostic ability as it leads to more appropriate treatment options.

Pressurization was effective in increasing the S/N ratio of TEOAEs in G2, but in G3, we infer that other issues, such as a change in the middle ear due to the presence of fluid, affected the capture of TEOAEs, which are not influenced by pressurization. For these newborns, referral for retesting is necessary. In such cases, it is important to inform parents about possible middle ear conditions as well as the possibility that there might be an inner ear defect, noting that the absence of OAEs in both tests is still not definitive, even if absorbance values are low.

In any case, absence of TEOAEs, even with normal absorbance values, can guide the examiner to think more critically regarding the referral of the newborn since normal middle ear conditions and absence of OAEs usually point to an inner ear alteration. Thus, a newborn with these conditions should be referred directly for audiological diagnosis, without going through the retest stage (since a FAIL result in the TEOAE would probably be confirmed). We conclude that, in NHS, it is important to include a test measuring the condition of the middle ear, preferably one evaluating absorbance values.

### 4.1. Comparison Between Groups

With regard to WBA, a comparison of the results between groups under the AP condition showed there was a statistically significant difference (*p* < 0.05) between groups G1 and G2 at frequencies 2, 3, and 4 kHz and between groups G1 and G3 at all frequencies. However, there was no significant difference between groups G2 and G3. Figure 7 shows that G2 presents intermediate values for all frequencies, with G1 having higher WBA and G3 lower WBA.

Comparing TEOAE S/N values between groups under the AP condition, there was a statistically significant difference (*p* < 0.05) between groups G1 and G2 and between groups G1 and G3 in all frequency bands. Under AP conditions, G1 was the only group with a PASS result. In groups G2 and G3, PASS results occurred only in the frequency bands of 3.97 and 4.97 kHz. Figure 8 shows that, in the same way as with WBA, G2 also presented intermediate values, with G1 having higher values and G3 having lower values.

Turning now to the results obtained at PP, there was a statistically significant difference (*p* < 0.05) in WBA between the groups: between groups G1 and G2 at frequencies of 2, 3, and 4 kHz, and between groups G1 and G3 at frequencies of 2, 3, 4, and 6 kHz. This is similar to what was observed at AP, which leads us to conclude that pressurization affected all groups equally (although for groups G2 and G3, only for 2 kHz). Figure 9 shows that, as at AP, the values of G2 are intermediate, with G2 values less than G1 values and G3 values less than G2 values.

Finally, when comparing TEOAE S/N results between groups under the PP condition, a statistically significant difference (*p* < 0.05) was observed between groups G1 and G2 at frequencies of 2, 3, 4, and 6 kHz. Although G2 had TEOAE S/N values elevated by pressurization while obtaining a PASS result, the values were still lower than in G1. Between groups G1 and G3 and between G2 and G3, a statistically significant difference (*p* < 0.05) was observed in all frequency bands tested, with lower values in G3 (Figure 10). This is because, after pressurization of the EAM, the newborns in group G2 (11.9%) who had failed the AP, obtained higher TEOAE values, with only newborns from G3 (19.5%) remaining in the FAIL group. Analyzing the results of absorbance and S/N of TEOAE, we observed that the WBA values at AP increased after pressurization (PP) in all groups and at most frequencies, which shows us that pressurization was able to increase absorbance in all ears. The increase in WBA from one condition to the other (AP and PP) can be seen in the S/N values of TEOAE, which also increased after pressurization in all groups but with a greater increase in G2, which also presented higher levels of increase in WBA values.

In addition, we noted that G2 (FAIL/PASS) presented intermediate values between the other two groups, with G1 (PASS/PASS) always having higher values and G3 (FAIL/FAIL) having lower values, both for WBA and S/N ratios of TEOAE. This fact leads us to believe that the newborns in G2, having presented lower WBA values than G1 at AP, presented a failure result in the TEOAE under this testing condition; on the other hand, the WBA values in G2 were not as low as in G3 since, after pressurization, their WBA values increased, which led to a PASS result under the PP condition. This did not happen with G3: although their WBA values also increased after pressurization, these values were so low at AP that the pressurization was not sufficient to cause the newborns to present a PASS result at PP.

This leads us to confirm that the middle ear changes presented by the ears in G3 should be related to other issues that went beyond a pressure change, as observed in G2. Finally, the increase in WBA values in G1 also resulted in an increase in TEOAE S/N values; however, this was already a group with high AP values, presenting a PASS result since the first test.

Studies on the application of OAE at peak pressure in newborns are scarce, with the exception of the study by Beck et al. [20], which deals with the application of DPOAE, and the study by Zimatore et al. [15], which evaluated TEOAE in 294 newborn ears, using the same parameters as the current study. However, Zimatore et al. did not find any FAIL results, except for reasons of high noise due to the movement of the newborns, and concluded that pressurizing TEOAE generates responses with relatively higher S/N ratios in the range of 0.87 to 4.97 kHz. In the same way as our data, they confirmed that pressurization was effective in increasing TEOAE S/N values in newborns with altered ME pressure, allowing them to be included in the PASS group after pressurization.

### 4.2. Final Considerations

In our study, we found that WBT was effective in identifying conductive alterations in newborns, and when used in conjunction with pressurization of the EAM, the technique can be useful for improving the capture rates of TEOAE. Therefore, in cases where there is a failure in an OAE result, together with a decrease in absorbance, it is suggested that the conditions of the middle ear be analyzed using WBT, and TEOAE recaptured under the PP condition. Such a testing method can eliminate the need to return for a retest. A reduction in retest rates will help to reduce anxiety in parents and avoid not having a follow-up for newborns who do not return for reevaluation.

We therefore suggest the following:

- Introducing WBT measurements in NHS programs whenever possible;
- That newborns who fail the initial NHS be reevaluated before discharge by means of pressurized TEOAE;
- Newborns who PASS the screening after pressurization should be discharged from the NHS, together with instructions about treating middle ear conditions;
- Newborns who continue to fail the screening after pressurization should be evaluated in terms of their middle ear conditions, and if adequate absorbance values are observed, they should be referred directly for audiological diagnosis.

We also emphasize the need for similar studies to be carried out on larger samples in order to improve knowledge about the technology of pressurized TEOAE in newborns.

## 5. Conclusions

Based on the analysis of the results of transient otoacoustic emissions at ambient pressure, it was observed that pressurization of TEOAE is effective in reducing the number of failures in neonatal hearing screening, making it possible to reduce the need for newborns to return for a retest.

## Figures and Tables

**Table 1:** Sample distribution considering right and left ears and male and female gender.

Gender	RE		LE		Total
	*N*	%	*N*	%	*N*
Male	51	55.4	51	54.8	102
Female	41	44.6	42	45.2	83
Total	92	100	93	100	185

**Table 2:** Newborns (ears) according to the result of the TEOAE at AP.

Ears	TEOAE-AP		
	PASS	FAIL	Total
*N*	127	58	185
%	68.5	31.4	100

**Table 3:** Means and standard deviations of S/N ratios for TEOAEs in each group for the AP condition.

Frequency (kHz)	0.87		1.94		2.96		3.97		4.97	
	Mean	SD	Mean	SD	Mean	SD	Mean	SD	Mean	SD
PASS	10.4	5.82	19.9	5.13	16.8	5.85	13.9	6.33	8.7	6.98
FAIL	1.80	4.54	0.9	6.83	0.1	5.90	0.8	3.76	−3.5	3.82
*p*-value	<0.001		<0.001		<0.001		<0.001		<0.001	

## Data Availability

The raw data supporting the conclusions of this article will be made available by the authors on request due to privacy concerns.
